# Using Kane’s validity framework to examine the implications of feedback in simulation-based assessments

**DOI:** 10.1186/s41077-025-00375-x

**Published:** 2025-10-21

**Authors:** Kathryn Hodwitz, Sherryn Rambihar, Gillian Nesbitt, Ara Tekian, Ryan Brydges

**Affiliations:** 1 Li Ka Shing Knowledge Institute, St. Michael’s Hospital, Unity Health Toronto, Toronto, ON Canada; 2 The Institute for Education Research, University Health Network, Toronto, ON Canada; 3 Mackenzie Health, Toronto, ON Canada; 4 Division of Cardiology, Department of Medicine, University of Toronto, Toronto, ON Canada; 5 Mt. Sinai Hospital, Sinai Health Systems, Toronto, ON Canada; 6 Department of Medical Education (DME), University of Illinois at Chicago, Chicago, IL USA; 7 College of Medicine, University of Illinois at Chicago, Chicago, IL USA; 8 St. Michael’s Hospital, Unity Health Toronto, Toronto, ON Canada; 9 Department of Medicine, University of Toronto, Toronto, ON Canada

**Keywords:** Validation, Feedback, Simulation, Assessment for learning

## Abstract

**Background:**

Simulation modalities have been increasingly used within programmatic assessment systems, yet educators typically have not collected and appraised validity evidence to justify such uses. Kane’s validity framework offers a contemporary approach to conducting validation studies of assessment practices. Under the framework, educators collect and appraise validity evidence according to four inferences: the scoring of performance, the generalization of scores to other assessment contexts, the extrapolation of assessment performance to real-world contexts, and the implications or consequences of assessment decisions for learners, educators, programs, patients, and society. We developed a simulation-based echocardiography competence assessment tool (ECAT) and collected validity evidence to evaluate its use as an assessment for learning. We applied Kane’s validity framework to evaluate the utility of the ECAT, with a focus on the implications of the assessment for promoting trainees’ learning.

**Methods:**

We implemented the ECAT in 2017, collecting simulation-based performance data and subsequent interview data. Fourteen cardiology trainees were assessed using the ECAT by four raters, and their performance was video-recorded. After trainees reviewed their performance videos and feedback, we conducted individual interviews with them and the raters who provided feedback. Directed content analysis generated implications and scoring evidence, and quantitative analyses generated scoring and extrapolation evidence. All evidence was critically appraised to form a validity argument about using ECAT as an assessment for learning.

**Results:**

Participants reported that ECAT scores accurately represented trainees'performance, and that the feedback helped identify learning opportunities. Inter-rater reliability was high at ICC = 0.913 (95% CI 0.81–0.97). Participants’ ECAT scores correlated with their end-of-rotation cardiology exam scores (*r* = 0.66, *p* = 0.02) and had positive associations with raters’ judgments of the diagnostic quality of their scans, and with their reported numbers of echocardiograms seen, performed, and interpreted.

**Conclusions:**

Our integrated analysis produced a data-informed validity argument supporting the use of the ECAT as a simulation-based assessment for learning. The findings also highlighted multiple areas for further research to optimize the ECAT. Our illustrative example of Kane’s validity framework aims to support simulation educators as they are increasingly called on to justify the use of simulation-based assessments in programmatic and competency-based assessment systems.

## Introduction

Assessment for learning and feedback remain central in most approaches to programmatic assessment [1]. Demands for assessments, and the data they generate, have increased in contemporary medical education, leading educators to increasingly consider simulation to provide more opportunities for hands-on learning, feedback, and assessment data [2–6]. To justify the resource investment and effectiveness of these assessments, evidence must be systematically collected to support using them to promote learning and make decisions about learners [7, 8].

Kane’s [9] framework for building validity arguments emphasizes collecting and critically appraising validity evidence across four inferences. These inferences include scoring (the degree to which assessment scores and feedback accurately represent performance), generalization (the degree to which the selected assessment stations and items represent the “universe” of possible assessment environments), extrapolation (the extent to which assessment performance predicts real-world performance), and implications (the consequences of the assessment decisions for learners, instructors, and society) [10, 11]. Kane’s framework enables program directors and educators to emphasize certain inferences over others and to collect evidence in different ways, given the unique purpose and stakes of their assessment program [11]. Notably, researchers in medical education have typically viewed these inferences as sequenced, interconnected steps, where each inference must be “fulfilled” before shifting to the next one. By contrast, we propose that the sequence and depth of data collection relating to each inference depend more on the assessment program’s validity argument for how and why the assessment data are being generated [10].

Validity frameworks have been explicitly supported in the standards for assessment [12], yet systematic reviews suggest they are underused in medical education [4, 13]. This may be due to a lack of practical examples for how to apply validity frameworks to evaluate assessment programs [8, 10, 14, 15]. Further, studies that use validity frameworks tend to report easy-to-generate validity evidence; conversely, implications evidence, arguably the most important and most difficult to obtain, has been rarely collected and reported [4, 16, 17].

The defensibility and utility of any assessment process hinge on regularly demonstrating supportive validity evidence. Scholars have noted a “social imperative” to demonstrate the validity of assessments in health professions education for both learners and patients, and consequently, a need to thoughtfully consider and evaluate the consequences or implications of a given assessment activity [18, 19]. Assessments for learning, for example, require educators to attend to the quality of feedback provided through an assessment (i.e., Kane’s scoring) and to how useful the recipients believe the feedback will be for their learning and subsequent practice change (i.e., Kane’s implications). Hence, when an assessment generates feedback to intentionally enhance trainees’ learning, educators could investigate whether trainees perceive the feedback (i.e., numerical scores and/or narrative comments about one’s performance) to be relevant, timely, actionable, and credible enough to identify learning opportunities and modify their practices [20–23]. Relatively few studies examine the impact of assessment feedback on learner behaviours [24], and fewer still evaluate it in the context of an overall validity argument.

We generated validity evidence for a simulation-based echocardiography competence assessment tool (ECAT) to evaluate its intended use of providing trainees with useful feedback for learning. We present our collection and appraisal of validity evidence to provide an example of how to formulate and evaluate a validity argument using Kane’s framework. We focused on echocardiography skills as an essential cardiology competency milestone [25] for which cardiology trainees may have insufficient workplace-based opportunities to develop competence, suggesting simulation-based assessments may be a useful supplement [26]. We focused on lower-stakes assessment, given that it may provide a useful starting place for program directors to build capacity in using Kane’s validation approaches before evaluating full assessment programs. Ultimately, we aimed to demonstrate the process of generating the claims and evidence for a validity argument with an emphasis on evaluating the implications of assessment feedback for learners.

## Methods

### Study overview

**Fig. 1:**
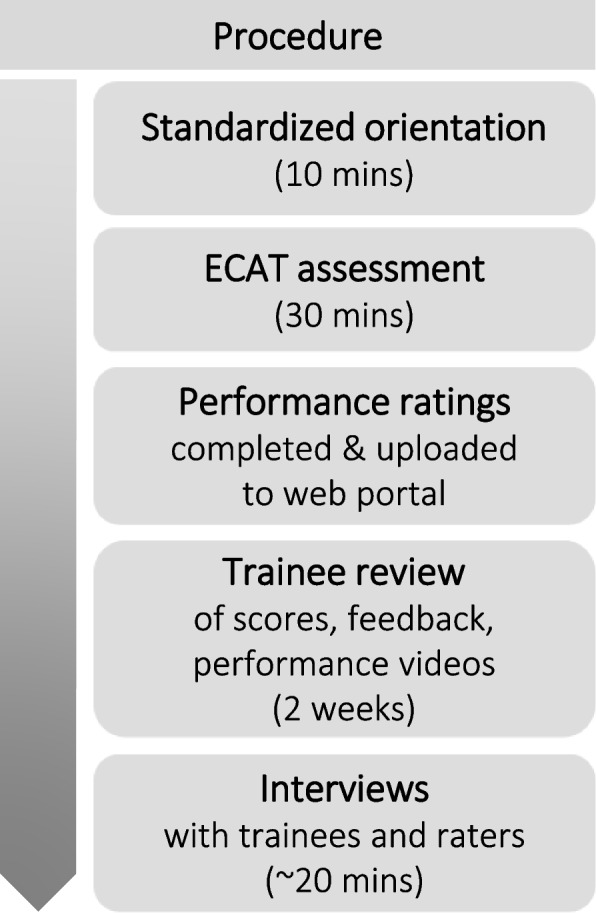
Study protocol and timeline

In 2017, we evaluated the use of the ECAT to provide feedback to cardiology trainees during simulation-based training of basic echocardiography skills [27]. Using a constructivist approach, we applied Kane’s framework to evaluate trainees’ and raters’ perspectives of feedback utility and to extrapolate ECAT scores to other programmatic assessment data. Data collection involved observing a single assessment, interviewing trainees and raters, and conducting psychometric analyses of tool performance (Fig. 1). We conducted this study in accordance with the Declaration of Helsinki and obtained ethics approval from the Toronto Academic Health System Network and the University of Toronto Office of Research Ethics. All participants consented to participate.

### Applying Kane’s validity framework

A validity argument begins with stating the assessment’s intended use and articulating specific claims or hypotheses for relevant inferences [10]. Educators then prioritize inferences based on the purpose and stakes of the assessment and iteratively collect and appraise data to determine whether to support the argument about the assessment’s intended uses.

We designed the ECAT to provide numerical scoring and narrative comments as feedback to cardiology trainees on basic echocardiography skills and to help them form a learning plan for further skill development. We developed specific interpretation/use arguments for each relevant validity inference (Table 1). We emphasized two primary inferences: (i) implications, framed as the degree to which the assessment would motivate learning and stimulate action towards practice change, and (ii) scoring, given that the accuracy and usefulness of the scores and feedback contribute to learners’ ability to develop data-driven learning plans. We also examined the relationship of the scores with other measures of clinical performance (extrapolation) and the consistency of scores across raters (generalization); however, we de-emphasized these inferences, given their relatively reduced importance for low-stakes assessments.

**Table 1:** Interpretation/use arguments and analytical plan for ECAT validity argument

Inference	Definition	Rationale for prioritization	Study interpretation/use argument	Methods and analyses
**Scoring**	The process of moving from observed performance relating to an underlying construct (e.g., diagnostic competence) to a single score describing that performance includes scoring rubrics, rules, procedures, and their translation into narrative or numerical data	Provides information about the accuracy and utility of the scores and feedback; an important precursor to examining if/how learners use the scores and feedback for learning (implications)	A tool can be developed based on a previously established tool to produce a more efficient and low-stakes ECAT	Independent analysis, criteria development, and team-based via consensus amongst three practicing cardiologists
			Learners will perceive the ECAT scores and feedback to be fair, accurate, and understandable	Content analysis of learner interviews
			Raters will perceive the ECAT to be useful for generating meaningful scores and feedback	Content analysis of rater interviews
**Implications**	The consequences of the assessment scores/comments for learners, teachers, and programs, including high-stakes decisions (e.g., pass/fail) and/or low-stakes actions (e.g., development of learning plans)	Provides important insight into the value of the assessment for learners, specifically the learning or practice change that is motivated by the feedback received	Learners will perceive the feedback and their own interpretations of performance data arising from the ECAT assessment to facilitate learning, stimulate practice change, and improve echocardiography skills	Content analysis of learner interviews
**Extrapolation**	The association of assessment performance with measures of real-world clinical performance, and/or established metrics in the training setting (e.g., exam scores, other competency rubrics)	Provides useful information about associations between ECAT to other existing assessments in the cardiology curriculum; however, a low-stakes purpose reduces the priority for such evidence	Learners’ ECAT scores will be positively associated with performance on the end-of-year echocardiography examination	Correlation between ECAT scores and end-of-year cognitive assessment scores
			Learners’ ECAT scores will be positively associated with the global impression of simulated echocardiogram diagnostic quality	Correlation between ECAT scores and determination of clinically relevant diagnostic quality
**Generalization**	The stability of the assessment output across different assessment contexts (i.e., across the universe of possible assessment scenarios)	We considered inter-rater reliability to be necessary and sufficient for the low-stakes implementation, where stability across the “universe of assessment” is not of high priority	Raters will use the ECAT consistently when scoring	Rater training, ICC coefficients

### Setting and participants

One author (SR) sent recruitment emails via program administrators to cardiology trainees from first to third year (*N* = 24) and to the senior echocardiography fellows from fourth or fifth year (*N*= 3). Participants attended the simulation session at the University of Toronto between June and July 2017. Our sampling strategy was based on convenience and previous literature for workplace-based assessment tools and observed structured technical assessments [28, 29]. Participants were not compensated.

### Tool development and training

#### Echocardiography Competence Assessment Tool (ECAT) development

We designed the ECAT by modifying a previous tool [27] for a simulation-based training setting (Appendix 1). The original tool was designed to provide feedback on trainees’ scanning skills and ability to identify anatomical structures in the clinical setting [27]. Given our use of the tool occurred away from the clinical setting, a team of three cardiologists (authors SR, GN, and a colleague) reduced the 88 items using the American, Society of Echocardiography’s recommended views (rather than Danish), removing items focused on advanced echocardiography, and based on limitations of the chosen simulator (CAE Vimedix, Montreal, Quebec). We called the resulting tool the ECAT, which contained 24 items corresponding to two essential questions for each of 12 standard echocardiography views: “Could participants angulate the probe to acquire the correct image?” and “Could they identify the necessary anatomical structures?” Notably, preliminary testing showed that the suprasternal echocardiography window was unattainable on the simulator; thus, this was excluded, leaving 11 standard echocardiography views and 22 total items. Such an extensive change to a tool necessitates “starting from scratch” when collecting validity evidence.

We changed the original scoring system from a 5-point Likert scale to a 4-point scale with descriptive behavioral anchors for competencies expected at different levels of residency training, which aligned with the American Society of Echocardiography endorsed scoring rubric: [30] not done = 0, expected performance by the end of training year 4 = 1, expected performance of training years 5–6 = 2, expected performance at end of training year 6 = 3. We added a global, binary determination of whether raters deemed the scan represented practical diagnostic quality. An added feedback component included two open-ended prompts, asking raters to document positive aspects of the scan and to suggest improvements.

#### Rater training

We recruited four board-certified echocardiography educators as raters. During a one-hour training session, raters: (i) observed videos of a first-year versus a senior cardiology fellow accessing three views on the simulator, (ii) independently scored each of the six clips using the ECAT, (iii) discussed areas of agreement and disagreement, and (iv) documented feedback they would provide, emphasizing specific performance aspects, suggestions for improvement, and using feedback for developing an action plan.

### Procedure and data collection

We collected demographic data, including level of cardiology training, number of previous echocardiograms seen, done, and interpreted, and number of previous general ultrasounds seen, done, and interpreted.

Participants individually completed the same standardized session (i.e., same location, equipment, staff, instructors, raters, and interviewers). Aligned with our assessment for learning focus, we provided each participant with a standardized, interactive orientation to ultrasound physics, standard echocardiographic views, and a high-fidelity echocardiography simulator (CAE Vimedix, Montreal, Quebec). We then provided an aid depicting the standard echocardiography examination with ideal images and anatomy, allowing 30 min for hands-on practice with access to a technical assistant.

Following the practice, we filmed participants’ performance focusing on the simulator screen while also capturing their hand movements during their investigations (while limiting capture of faces and other identifying information). The technical assistant prompted participants to produce the 11 standard echocardiography views, acquired a digital video loop of three consecutive cardiac cycles from the simulator, and prompted participants to verbally identify anatomic structures on the screen using a stylus. The assistant uploaded participants’ 11 de-identified echocardiography simulator video clips and 11 de-identified anatomy identification video clips to an encoded web portal for later access. For feasibility purposes, one on-site rater uninvolved in residency training performed real-time, in-person scoring, recorded their feedback for the portal, and did not provide verbal feedback. The remaining three raters reviewed de-identified video clips on the portal, assigned ratings, and recorded their feedback. We collated all four raters’ anonymous feedback for each participant.

Two weeks later, we conducted semi-structured interviews via telephone to understand if and how participants perceived the ECAT and whether feedback had stimulated learning or prompted changes in their practice behavior. We chose 2 weeks based on the relatively brief amount of time committed to the simulation-based assessment; longer delays could have resulted in any impacts of the assessment having been “washed out.” We also interviewed raters to examine their perspectives on whether the ECAT assisted them in scoring and providing feedback. Interviews were approximately 20 min in length. An independent third-party de-identified and transcribed the interview audio files verbatim.

### Analysis

Our analyses corresponded to each interpretation/use argument in Table 1. We analyzed participant demographic and quantitative performance data using Statistical Analysis Software version 9.2 (SAS Institute, Inc., Cary, NC). We computed test-item statistics including mean and standard deviation for the two dimensions assessed per echocardiography view. We assessed the tool’s internal consistency with coefficient alpha. We assessed inter-rater reliability using the intra-class correlation coefficient (ICC) computed as an item-specific result and overall.

Our univariate linear regression models assessed relationships between participants’ ECAT scores (the dependent variable) and their self-reported sex, level of training, and prior experiences with ultrasound and echocardiography. We used a generalized estimating equation model to find the association between ECAT score and the raters’ binary determination of diagnostic quality. We assessed the Pearson correlation between the average ECAT score and participants’ end-of-year echocardiography exam (which occurred ~ 1 month after training). For all assessments, we considered an alpha level of *p* < 0.05 as significant.

We coded the interview data both inductively and deductively, identifying codes as they arose, and grouping codes into the inferences in Kane’s model, as appropriate. Initially, two investigators (S.R. and R.B.) independently coded two randomly selected transcripts and reviewed each other’s codes to develop a draft coding framework. A third investigator (K.H.) then coded all transcripts using the coding framework, iteratively revising the coding framework and seeking input from the team throughout analysis. Our audit trail included meeting notes relevant to individual preliminary coding, grouping of codes into themes, and selecting representative quotes.

Upon analyzing the qualitative and quantitative data, we reviewed the findings holistically and integrated them conceptually to formulate a validity argument, as expected in Kane’s framework. For example, after coding all interview data pertaining to raters’ perceived effectiveness of the scoring tool and trainees’ perceived value of the resulting scores and feedback, our team met to critically appraise the data together to formulate a data-informed argument for the scoring inference. Similarly, the team appraised the interview data pertaining to trainee’s perceptions of whether and how the scores and feedback influenced their learning or behaviour to formulate an argument for the implications inference. Collectively, we appraised all the evidence to ascertain which inferences were supported and where further refinement and continued evaluation would be needed.

Throughout data generation and analysis, we reflexively acknowledged how our professional roles, personal perspectives, and prior knowledge may have influenced the research process. Collectively, our team has extensive knowledge in cardiology, self-regulated learning, assessment, feedback, and validity theory, which informed our analyses and interpretation of findings.

## Results

Table 2 provides an overview of how we integrated the findings across our various data sources to inform how we formulated inference-specific and an overall validity argument.

**Table 2:** Integrated findings informing our validity argument

Inference	Interpretation/use arguments	Data sources and findings	Overall evaluation of validity evidence
Scoring	A tool can be developed based on a previously established tool to produce a more efficient and low-stakes ECAT	Our process led to the 22-item ECAT for use with the simulator in this study	Packaged as an efficient and relatively brief tool, the ECAT scoring appears to be accurate and useful for both learners and raters in the context of an assessment for learning. Some attention to post hoc video assessments during rater training may be needed to support future scoring
	Learners will perceive the ECAT scores and feedback to be fair, accurate, and understandable	Participants reported the ECAT scores to be a fair and accurate representation of their performance	
	Raters will perceive the ECAT to be useful for generating meaningful scores and feedback	Two of three raters stated the tool helped them translate performance into scores. One rater found the post hoc video assessment modality challenging	
Implications	Learners will perceive the feedback and their own interpretations of performance data arising from the ECAT assessment to facilitate learning, stimulate practice change, and improve echocardiography skills	Participants reported that the assessment feedback identified learning opportunities and motivated them to improve their knowledge or skills Access to the online portal and feedback from multiple raters were seen as beneficial for learning We observed an interaction between training stage and assessment utility, with “advanced beginner” trainees reporting greater value compared to novice and senior trainees	ECAT feedback is perceived as valuable for identifying learning opportunities and motivating trainees’ self-reported future learning plans Additional coaching for very novice trainees and added challenge for more senior trainees may help to enhance the implications of assessment feedback for students across the continuum of expertise
Extrapolation	Learners’ ECAT scores will be positively associated with performance on the end-of-year echocardiography examination	Correlation between participants’ ECAT score and end-of-year examination score: *r* = 0.66 (*p* = 0.02)	ECAT performance is associated with other relevant measures of clinical performance, supporting extrapolation
	Learners’ ECAT scores will be positively associated with the global impression of simulated echocardiogram diagnostic quality	ECAT performance was associated with: level of training (16.7 (4.9), *p* = 0.005), number of echocardiograms seen (27.6 (3.1), *p* = 0.014), number of echocardiograms performed (0.06 (0.02), *p* = 0.012), and number of previously completed and directly interpreted echocardiograms (0.02 (0.01), *p* = 0.024)	
Generalization	Raters will use the ECAT consistently when scoring	Successful rater training, and ICC = 0.91 (95% CI 0.81–0.97), indicating strong inter-rater reliability	The ECAT appears to produce stable scores when the same learners have their performance measured by different raters

### Participants

Our cohort included five first-year trainees, five second-year trainees, and four third-year trainees or postgraduate fellows. Six of 14 (43%) were female. The four raters represented early career (< 5 years in practice), mid-career (> 10 years), and late-career (20 + years) cardiologists (*N* = 3) and a sonographer (*N* = 1). Three of the four were female. Three raters participated in interviews, and one functioned as the interviewer (author SR).

### Scoring evidence

For scoring evidence, we analyzed how participants perceived the accuracy and usefulness of the scores and feedback from the assessment. Participants described the assessment as being fair, accurate, and representative of their performance: “it did match with my impressions of the day of the performance” (P1).

By contrast, the raters had varying perceptions of the scoring process. Two appreciated that the scoring rubric’s objectivity helped them to provide concrete, constructive feedback to learners: “it allowed me to formulate their performance… with a very practical approach of not only where they are at now, but what they could do moving forward to improve” (R1). Conversely, the third rater articulated challenges in providing feedback, given they did not observe the trainee in person: *“*You couldn’t see, when they were doing their images, what they struggled through or what they didn’t… it has to be real-time feedback, not recorded feedback.” Although not directly related to the tool, this challenge in providing feedback based on recorded rather than real-time performance highlights a potential challenge for raters using ECAT and the chosen simulator.

### Implication evidence

For implication evidence, we first considered the psychological impacts of the assessment. Most participants reported experiencing the assessment as comfortable, non-stressful, and conducive to learning: “I didn’t see it as like an exam… or that I was being watched… I definitely think it helped learning.” (P10).

Almost all participants indicated that the feedback they received stimulated learning and motivated action to improve their knowledge or skills. They described the feedback as helping to isolate specific areas for improvement and to inform self-regulated learning, noting that the feedback highlighted learning opportunities and “gaps in knowledge that would otherwise not [have been] identified” (P11).

[The feedback] made me go back to first principles and look up things in textbooks to try and identify more of the anatomy and structures in preparation to perform the procedure. P1

Further, participants suggested that having access to their performance data through the web portal would facilitate their ongoing learning. Some indicated that they had already accessed this data for learning, and may continue to use it as a resource:

It was nice to have the ability to look at my videos as well, just as a… reminder of what I did at that time. And having the feedback, and saying, what I should do in these views, it would help me correlating that piece back with the videos that I still have. P12

Receiving feedback from multiple raters was reportedly beneficial for learning, in that it: “reinforced that if all people noticed it then it’s probably something I could actually think about and use.” (P9). Notably, the source of feedback (physician vs. sonographer) did not influence perceived credibility, as participants valued and appreciated receiving both perspectives.

It’s nice to get feedback from sonographers and staff just because they do have different expertise and different advice to give depending on that expertise… I think it was more beneficial to me, in the end. P12

Not all participants found the feedback useful for formulating concrete learning plans, however. Some spoke generally about their feedback without recalling details, implying a defined action plan was unlikely: “I'm trying to remember what exactly the detailed feedback was, but… [I remember] thinking, you know, these are things that I should work on.” (P4). Training stage also appeared relevant, with some senior participants noting the feedback would have a “very minor” (P9) impact on their learning, though they suggested value for such an assessment earlier in their training:

If I were still in the earlier part of the growth curve, I would have [used the feedback in practice]… if I were a trainee and I was still learning, I think it'd be very useful because… a lot of times, you don't have a good sense of what you're doing wrong. P14

That said, one junior participant found the feedback difficult to understand, which they attributed to their lack of prior echocardiography knowledge:

This feedback was a little bit hard to interpret. There was a lot of technical information in it that I don’t necessarily know what to do with improving my skill set versus if someone told directly, “this is what you’re doing wrong, move it this way. P3

Overall, many participants noted valuing the assessment and the opportunities to improve their echocardiography skills.

Echo is something that’s a huge learning curve for us but it’s so, so important and spans all parts of cardiology… So it has to be something that’s foundational and taught early on. And there has to be some form of standardization to the process. P6

### Extrapolation evidence

Most analyses for the extrapolation inference were exploratory, given the limited sample size. After modeling “diagnostic quality” as a yes/no variable, both statistical models suggested a significant difference in participants’ average ECAT score when the diagnostic quality was judged as “yes” vs. “no,” with higher scores associated with “yes.” Using the linear model, *F*-statistic was 20.26 (*p* = 0.0007).

We had data for 12/14 (86%) participants’ end-of-year echocardiography examinations, with two postgraduate fellows excluded from sitting the exam. The correlation between participants’ ECAT score and end-of-year examination score was meaningful at *r* = 0.66 (*p* = 0.02); although the two assessments did not occur contemporaneously, we believe this correlation demonstrates initial supportive extrapolation evidence. Our univariate linear regressions exploring associations between participants’ self-reported variables and their ECAT scores showed meaningful relationships for level of training (*t*_*1,13*_ = 3.98, standardized *β* = 0.75, *p* = 0.002), number of echocardiograms seen (*t*_*1,13*_ = 2.87, standardized *β* = 0.64, *p* = 0.014), number of echocardiograms performed (*t*_*1,13*_ = 2.95, standardized *β* = 0.65, *p* = 0.012), and number of previously directly interpreted echocardiograms (*t*_*1,13*_ = 2.58, standardized *β* = 0.60, *p* = 0.024). All associations for self-reported sex and number of ultrasounds seen, performed, and interpreted were not statistically significant.

### Generalization evidence

To demonstrate the benefits of selecting the 24-item ECAT items via consensus amongst three practicing cardiologists, we found that the internal consistency across all ECAT items was 0.84 (95% CI, 0.81–0.87). We also found strong inter-rater reliability, with an ICC = 0.91 (95% CI, 0.81–0.97). See Appendix 1 for participants’ mean scores on the two dimensions assessed for the 11 echocardiography views.

## Discussion

We sought to demonstrate how to use Kane’s validation framework to collect and appraise validity evidence for a simulation-based assessment. We found that, overall, raters used the ECAT consistently and reported that it facilitated their scoring and feedback. Trainees found these scores to be fair, accurate, relevant, and actionable, and described intending to use the feedback and performance videos to inform future self-regulated learning activities. Our analyses also demonstrated meaningful relationships between participants’ ECAT scores and their prior level of echocardiogram experience, and an end-of-year exam. Aggregating this evidence, we offer the following validity argument: when using this ECAT and the chosen simulator to stimulate assessment for learning, educators can be confident that raters will provide actionable feedback that informs most trainees’ learning plans. However, some junior trainees will likely need additional coaching to interpret the feedback, whereas some senior trainees would need more challenging tasks.

We formulated our validity argument by closely appraising data across inferences and generating three areas for further evaluation (see Table 2). Firstly, although the ECAT seemed ideally suited for advanced beginners with some prior echocardiography knowledge, educators would likely need to tailor it to benefit senior trainees (e.g., increasing case complexities) and relatively novice trainees (e.g., avoiding technical jargon in feedback). Researchers could study how best to tailor the assessment stimulus (i.e., simulator views and requirements), available educational supports (e.g., worked examples) [5], and rater processes (e.g., feedback specificity and type) [31] to each trainee’s level of prior knowledge. Secondly, comparing the impacts of post hoc video versus in situ direct rater observation appears relevant, given one rater’s expressed difficulty providing feedback based on video. Although low-stakes settings afford the increased use of video-based assessments, which can improve reliability and reduce rater bias [32, 33], such assessments may also require additional training to increase raters’ skill [34]. Thirdly, synthesizing our implications evidence suggests the ECAT provided greater support for trainees’ perceived immediate learning than for their subsequent practice change, which may be related to the short proximity between the ECAT experience and our subsequent interviews. However, this finding may also reflect the broader challenge of promoting and measuring changes to future practice following assessment. Researchers must continue to try to resolve the “wicked problem” of linking assessments, trainees’ subsequent learning activities, and their future practice behaviours [35].

Rather than an endpoint, our validity argument represents a step in an ongoing process [9–11]. We prioritized scoring, implications, and extrapolation evidence, meaning future validation efforts must focus on generalization evidence and continued appraisal as uses of the ECAT change. We sought to provide educators and administrators with a worked example of how to conduct a validation study and, specifically, how to collect implication evidence for low-stakes assessments. In terms of the workload this requires of program directors, we believe we have demonstrated how relatively typical data collection and analysis methods, used with a relatively small number of participants, can yield valuable, informative, and actionable validity evidence. As simulation continues to be used as a method of providing feedback to learners, it is critical that educators systematically evaluate the extent to which that feedback promotes learning and practice improvement.

### Strengths and limitations

Study strengths include our use of multiple raters, multiple analysts, and the strategic collection of quantitative and qualitative data sources to inform our validity argument. Our small sample size resulted in reduced statistical power for the quantitative analyses; however, this initial study allowed us to generate sufficient evidence to develop a relatively robust validity argument. The gap between the simulation session and the end-of-year exam may mean that additional intervening factors contributed to the observed correlation. Although our sample included learners from different levels of expertise, it represented a convenience sample and future studies could recruit learners more systematically to understand the influence of other personal or contextual factors on the implications of such assessments. Multiple factors contributed to the delayed timeframe from study to publication (e.g., change in author institutions and professional roles, the pandemic), which may be seen as a limitation; however, we argue that the findings remain relevant, and the study provides a useful template for others wishing to systematically collect validity evidence for assessments in medical education. As simulation modalities and educational technologies continually advance, we believe that Kane’s validity framework remains a versatile system that educators can use to develop and test validity arguments across assessment contexts.

## Conclusions

Our validity argument demonstrates the preliminary value of ECAT for low-stakes use among advanced-beginner trainees. Our worked example of applying Kane’s framework may help educators within and outside cardiology to develop and critically evaluate simulation-based assessments. As programmatic assessment prompts increased demands for data, we believe educators must demonstrate how each assessment promotes learning and supports decision-making using established frameworks. Systematically collecting evidence for the implications of assessments for learning is particularly important for promoting trainees’ workplace-based learning and stimulating practice change.

## Data Availability

No datasets were generated or analysed during the current study.

## Appendix 1

**Table 3:** ECAT item-level descriptive statistics

Variable	Mean (standard deviation)
Apical two-chamber window use of screen	0.9 (0.8)
Apical three-chamber window use of screen	1.0 (1.0)
Parasternal short-axis mitral valve window use of screen	1.1 (1.1)
Apical five-chamber window use of screen	1.1 (0.9)
Right ventricular inflow window use of a screen	1.2 (1.1)
Parasternal short-axis papillary muscle window use of screen	1.2 (1.0)
Apical four-chamber window use of screen	1.2 (0.9)
Parasternal long axis window use of screen	1.2 (0.8)
Parasternal short-axis aortic valve window use of screen	1.2 (0.8)
Parasternal short-axis apex window use of screen	1.3 (1.2)
Right ventricular inflow window anatomy identification	1.3 (1.0)
Apical three-chamber window anatomy identification	1.3 (1.0)
Apical five-chamber window anatomy identification	1.3 (0.6)
Parasternal short-axis mitral valve window anatomy identification	1.9 (0.9)
Parasternal short-axis aortic valve window anatomy identification	1.5 (1.1)
Parasternal short-axis papillary muscle window anatomy identification	2.0 (0.9)
Subcostal window use of screen	1.5 (1.1)
Apical four-chamber window anatomy identification	1.5 (0.6)
Apical two-chamber window anatomy identification	1.7 (0.9)
Parasternal long axis anatomy identification	2.0 (0.9)
Subcostal window anatomy identification	2.0 (0.8)
Parasternal short-axis apex window anatomy identification	2.6 (1.0)

We report the mean score (standard deviation) for each item, where the minimum potential score was 0 and the maximum potential score was 3
